# Immunologic and virologic failure after first-line NNRTI-based antiretroviral therapy in Thai HIV-infected children

**DOI:** 10.1186/1742-6405-8-40

**Published:** 2011-10-26

**Authors:** Torsak Bunupuradah, Thanyawee Puthanakit, Pope Kosalaraksa, Stephen Kerr, Pitch Boonrak, Wasana Prasitsuebsai, Pagakrong Lumbiganon, Tawan Mengthaisong, Chayapa Phasomsap, Chitsanu Pancharoen, Kiat Ruxrungtham, Jintanat Ananworanich

**Affiliations:** 1 The HIV Netherlands Australia Thailand Research Collaboration (HIV-NAT), The Thai Red Cross AIDS Research Center, Bangkok, Thailand; 2 Department of Pediatrics, Faculty of Medicine, Chulalongkorn University, Bangkok, Thailand; 3 Srinagarind Hospital, Khon Kaen University, Khon Kaen, Thailand; 4 Kirby Institute for Infection and Immunity in Society, UNSW, Sydney, Australia; 5 Department of Medicine, Faculty of Medicine, Chulalongkorn University, Bangkok, Thailand; 6 SEARCH, The Thai Red Cross AIDS Research Center, Bangkok, Thailand

**Keywords:** pediatric HIV, NNRTI-based HAART, treatment outcome, virologic failure

## Abstract

**Background:**

There are limited data of immunologic and virologic failure in Asian HIV-infected children using non-nucleoside reverse transcriptase inhibitor (NNRTI)-based highly active antiretroviral therapy (HAART). We examined the incidence rate of immunologic failure (IF) and virologic failure (VF) and the accuracy of using IF to predict VF in Thai HIV-infected children using first-line NNRTI-based HAART.

**Methods:**

Antiretroviral (ART)-naïve HIV-infected children from 2 prospective cohorts treated with NNRTI-based HAART during 2001-2008 were included. CD4 counts were performed every 12 weeks and plasma HIV-RNA measured every 24 weeks. Immune recovery was defined as CD4%≥25%. IF was defined as persistent decline of ≥5% in CD4% in children with CD4%<15% at baseline or decrease in CD4 count ≥30% from baseline. VF was defined as HIV-RNA>1,000 copies/ml after at least 24 weeks of HAART. Clinical and laboratory parameter changes were assessed using a paired t-test, and a time to event approach was used to assess predictors of VF. Sensitivity and specificity of IF were calculated against VF.

**Results:**

107 ART-naive HIV-infected children were included, 52% female, % CDC clinical classification N:A:B:C 4:44:30:22%. Baseline data were median (IQR) age 6.2 (4.2-8.9) years, CD4% 7 (3-15), HIV-RNA 5.0 (4.9-5.5) log_10_copies/ml. Nevirapine (NVP) and efavirenz (EFV)-based HAART were started in 70% and 30%, respectively.

At 96 weeks, none had progressed to a CDC clinical classification of AIDS and one had died from pneumonia. Overall, significant improvement of weight for age z-score (p = 0.014), height for age z-score, hemoglobin, and CD4 were seen (all p < 0.001). The median (IQR) CD4% at 96 weeks was 25 (18-30)%. Eighty-nine percent of children had immune recovery (CD4%≥25%) and 75% of children had HIV-RNA <1.7log_10_copies/ml.

Thirty five (32.7%) children experienced VF within 96 weeks. Of these, 24 (68.6%) and 31 (88.6%) children had VF in the first 24 and 48 weeks respectively.

Only 1 (0.9%) child experienced IF within 96 weeks and the sensitivity (95%CI) of IF to VF was 4 (0.1-20.4)% and specificity was 100 (93.9-100)%.

**Conclusion:**

Immunologic failure, as defined here, had low sensitivity compared to VF and should not be recommended to detect treatment failure. Plasma HIV-RNA should be performed twice, at weeks 24 and 48, to detect early treatment failure.

**Trial Registration:**

**Clinicaltrials.gov identification number **NCT00476606

## Background

Over 140,000 children in South and Southeast Asia are living with HIV [1]. Treatment with highly active antiretroviral therapy (HAART) has increased the life expectancy of HIV-infected children [2-6]. Non-nucleoside reverse transcriptase inhibitor (NNRTI)-based HAART is commonly prescribed as the first-line regimen in resource-limited settings [2-8]. In Thailand, the majority of HIV-infected individuals have HIV-1 subtype CRF01_AE [9]. A national program has provided free HAART since 2004 [2,3].

HIV care and monitoring in resource-limited settings rely on clinical and immunologic monitoring. Virologic monitoring is not commonly used due to the high cost and limited access. Current WHO HIV treatment guidelines in resource-limited settings do not recommend routine plasma HIV-RNA measurement, rather they suggest using immunologic criteria for making decisions about treatment failure [10]. Mee et al. reported low sensitivity of CD4 criteria of 21.2% in detecting virologic failure in African HIV-infected adults [11]. Immunologic failure criteria have poor accuracy and are estimated to lead to premature switching to second-line regimens for 43-58% of HIV-infected adults who experience a decrease in CD4 cell count [12]. Reports in African HIV-infected children have shown low sensitivity of 3.5% of clinical and immunologic criteria based on the WHO criteria for IF available at that time [2007] in identifying virologic failure [13].

There are limited data on the sensitivity and specificity of immunologic compared to virologic failure in Asian HIV-infected children. Therefore, we aimed to report the incidence rate of virologic failure and immunologic failure and the accuracy of using immunologic failure to predict virologic failure within 96 weeks of initiating first-line NNRTI-based HAART in HIV-infected children.

## Materials and methods

Data from HIV-infected children who were enrolled in 2 prospective cohorts at the HIV Netherlands Australia Thailand Research Collaboration (HIV-NAT), the Thai Red Cross AIDS Research Centre, Bangkok and Khon Kaen University, Khon Kaen, Thailand from December 2001 to March 2008 were used. The inclusion criteria for this study were HIV-infected children aged 1-18 years at enrolment who were antiretroviral therapy (ART) naïve before initiating first-line NNRTI-based HAART. The criteria to start HAART in these HIV-infected children followed WHO 2006 guidelines [14] and Thai 2008 guidelines [15]; CDC clinical classification C, or CD4% <20% in children aged < 3 years, or CD4%<15% in children aged ≥ 3 years [14,15]. The ART regimen and dosages were calculated according to a child's body weight or body surface area according to recommendations in the WHO 2006 guidelines [14] and Thai 2008 guidelines [15]. Adherence counselling was performed at each visit according to standard practice. The self-report adherence questionnaires were completed by caregivers at every visit. The missing doses in the last 3 days or missing > 15 doses since last visit were defined as poor adherence.

The children were assessed every 12 weeks for CDC clinical classification, weight and height measurements, complete blood count, CD4 percent and CD4 cell count and alanine transferase (ALT). Plasma HIV- RNA, Roche Amplicor Ultrasensitive assay, Palo Alto and USA were performed every 24 weeks from 2007. The plasma, which had been stored every 24 weeks during 2001-2006, was tested retrospectively for HIV-RNA and included in this analysis. Fasting lipid profiles and glucose were performed every 24 weeks since June 2006. Clinical and laboratory adverse events (AEs) were graded according to the Division of AIDS toxicity table 2004 [16]. The ethics committees at the institutions in both Bangkok and Khon Kaen approved the study, and all caregivers signed written informed consent.

### Definitions

Immune recovery was defined as recovery of CD4 to ≥ 25% after HAART initiation [17]. Immunologic failure (IF) was defined as persistent decline of ≥ 5% in CD4% in children with CD4% <15% at baseline or decrease CD4 count ≥ 30% from baseline [18]. Virologic suppression was defined as HIV-RNA <1.7 log_10_copies/ml. Virologic failure (VF) was defined as plasma HIV-RNA more than 1,000 copies/ml after 24 weeks of HAART initiation [5,19]. Elevated total cholesterol (TC), triglyceride (TG) and reduced high density lipoprotein (HDL) were defined as TC ≥ 200 mg/dl, TG ≥ 150 mg/dl and HDL < 40 mg/dl [20].

### Statistical analysis

Statistical analysis was performed with SAS version 9.1 (SAS Institute Inc, Cary, NC, USA) and with Stata version 11 (Statacorp, College Station, TX, USA). Changes in weight, height, and laboratory parameters from baseline to weeks 48 and 96 were calculated and a formal comparison using a paired t-test was made at week 96. Patients changing from NNRTI-based regimens to PI-based regimens for reasons other than virologic failure were censored at this point and no extrapolation of data was used. The Kaplan-Meier method was used to assess the proportion of patients with virologic failure and Cox Proportional Hazards regression was used to assess the relative risk of failure. Significance was assessed using a 2-sided p-value of 0.05.

## Results

One hundred and seven HIV-infected children were included in the analysis. The median (IQR) age was 6.2 (4.2-8.9) years, 52.3% were female. Zidovudine (AZT), and stavudine (d4T) were used in 60.4% and 39.6%, respectively. All used lamivudine (3TC). Nevirapine (NVP) and efavirenz (EFV)-based HAART were prescribed 70% and 30%, respectively. The median (IQR) baseline CD4% and HIV-RNA were 7 (3-15) and 5.0 (4.9-5.5) log_10 _copies/ml (Table 1).

**Table 1:** Baseline characteristics of children receiving first-line NNRTI-based HAART

Characteristics*	N	
Age, years	107	6.2(4.2-8.9)
Female,%	107	52.3
CDC clinical classification N:A:B:C,%	107	4:44:30:22
Weight for age z-score	107	-1.5(-2.4 to -0.4)
Height for age z-score	107	-1.7(-2.6 to -1.1)
Weight for height z-score	107	-0.5(-1.6 to 0.3)
Hemoglobin, g/dL	78	10.6(9.5-11.7)
CD4%	101	7(3-15)
CD4 count, cells/mm^3^	101	151(35-477)
HIV-RNA log_10 _copies/ml	100	5(4.9-5.5)
NVP:EFV-based HAART,%	107	70:30

CDC: Center for Disease Control and Prevention, NVP: nevirapine, EFV: efavirenz,

HAART: highly active antiretroviral therapy

*Data was shown in median (IQR) or percentage

During 96 weeks of follow-up, there were 2 deaths: one girl died in a car accident after 48 weeks and one boy died at week 12 due to pneumonia. No children had disease progression of CDC clinical classification. Improvement of median z-score of weight for age (p = 0.014) and height for age (p < 0.001) compared to baseline visit are shown (Table 2). For laboratory results, improvement of hemoglobin, CD4% and CD4 count were found (all p < 0.001). At 96 weeks, 89% of children had immune recovery and 75% had HIV-RNA < 1.7 log_10 _copies/ml (Table 2).

**Table 2:** Treatment outcomes during 96 weeks

Outcomes*	Week 0 N = 107	Week 48 N = 103	Week 96 N = 98	p-value
**Clinical outcomes**				
Weight for age z-score	-1.5(-2.4, -0.4)	-1.1(-1.8,-0.6)	-1.2(-2.0,-0.5)	0.014
Height for age z-score	-1.7(-2.6, -1.1)	-1.7(-2.3,-1.0)	-1.4(-2.0,-0.6)	<0.001
Weight for height z-score	-0.5(-1.6, 0.3)	-0.1(-0.6,0.5)	-0.3(-1.2,0)	>0.05
**Laboratory outcomes**				
Hemoglobin (g/dL)	10.6(9.5-11.7)	12.1(11.0-12.7)	12.1(11-12.7)	<0.001
CD4%	7(3-15)	21(15-25)	25(18-30)	<0.001
CD4 count (cells/mm^3^)	151(35-477)	531(367-811)	707(422-915)	<0.001
Percent of children with immune recovery (%)	0	82.2	89.1	
Percent of children with HIV-RNA<1.7log_10_copies/ml (%)	0	69.1	75.0	
ALT (U/L)	27(16-46)	22(16-28.5)	22(17-28)	0.01

Immune recovery was defined as recovery of CD4 to ≥ 25% after HAART initiation

*Data was shown in median (IQR)

**Figure 1:**
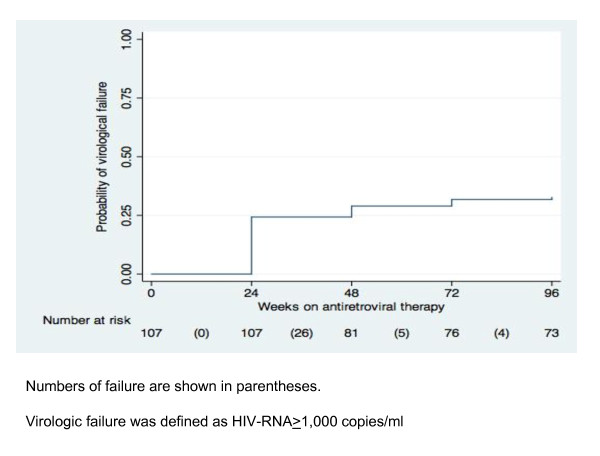
**Kaplan-Meier proportion of virologic failure**.

One child experienced IF and VF at week 24 and his CD4 was improved after switch to second-line therapy. Thirty-five children experienced VF within 96 weeks. Of these, 24 (68.6%) and 31 (88.6%) children had VF in the first 24 and 48 weeks respectively. A Kaplan-Meier graph of VF is presented in Figure 1.

During the study period, 17 (22.1%) of 77 children who had completed data of adherence questionnaires were noted to have poor adherence by history. Three of these 17 children had VF. There was no association between poor adherence by history and VF (p = 0.24).

### Predictors of virologic failure

In univariate Cox's regression to identify the predictor of VF, the hazard ratio (95% confidence interval) for gender, male versus female was 0.92 (0.47-1.80), age at NNRTI-based HAART initiation, > 6 years versus ≤ 6 years was 0.70 (0.36-1.37), CD4% at NNRTI-based HAART initiation, ≤ 7:versus > 7% was 1.08 (0.55-2.12), CDC clinical classification, C versus non C was 1.03 (0.47-2.27), NNRTI type, NVP versus EFV was 1.46 (0.66-3.22), HIV-RNA log_10 _at NNRTI-based HAART initiation, > 5 versus ≤ 5 was 1.35 (0.65-2.81). Since no factors were significant in univariate analysis, no multivariate model was developed.

### Sensitivity and specificity of immunologic failure

One child experienced IF at week 24. The sensitivity and specificity (95% confidence interval; CI) of IF to VF were 4 (0.1-20.4)% and 100 (93.9-100)%, respectively. Positive predictive value and negative predictive value were 100 (2.5-100)% and 71.1 (60.1-80.5)%, respectively.

### Severe adverse events

One girl aged 13 years had intermittent elevated ALT grade 3-4 from week 4 related to chronic hepatitis B infection. One boy aged 5 years with baseline hemoglobin 11.4 g/dL, initiated treatment with zidovudine/lamivudine/nevirapine and developed grade 4 anemia (hemoglobin 5.4 g/dL) at week 36. After receiving packed red cell transfusion he was switched to stavudine/lamivudine/nevirapine, the clinical condition was improved at week 48 with hemoglobin 12.1 g/dL.

At 96 weeks, 64 (60%) children had fasting TG and TC results and 40 (37%) children had fasting HDL results available. The median (IQR) TC was 187(157 - 212), TG was 84(68-110.5), and HDL was 62(46 - 79) mg/dL. The proportion of children with elevated TC was 32.8%, elevated TG was 7.8%, and reduced HDL was 6.3%.

## Discussion

At 96 weeks after NNRTI-based HAART initiation, HIV-infected children had significant improvement of growth, hemoglobin, CD4%, and CD4 count. Only 1 child experienced IF. VF mostly occurred within the first 24 weeks of NNRTI-based HAART but a quarter developed failure between 24 to 48 weeks. To detect early treatment failure, having two HIV-RNA monitorings within the first year, at weeks 24 and 48, would be ideal. Low sensitivity of IF to predict VF was found.

Growth was significantly improved after commencement of NNRTI-based HAART in HIV-infected children [3]. In our report, we found the same significant improvement of weight for age and height for age z-score as found in African HIV-infected children [21,22].

Immunologic status was significantly improved by NNRTI-based HAART and sustained over a long period (2, 3, 17). In our report, 89% of children had immune recovery at 96 weeks which is higher than the 50% reported in a study of children from the northern part of Thailand [3]. The children in our study were younger and had higher baseline CD4, which could account for the difference in outcome. The majority of HIV-infected children have undetectable HIV-RNA after NNRTI-based HAART initiation. The proportion of children with undetectable HIV-RNA in our report was comparable to previous publications as of 70-75% [23,24].

In our study, 33% of children experienced VF. Varying VF rates have been reported in other studies. Jittamala P. et al. reported VF in 20% of 202 Thai HIV-infected children, median duration of treatment until VF was 26 (range: 24-168) weeks [5]. Barth R. et al. reported VF in 38% of 81 HIV-infected children in rural South Africa with the median duration of NNRTI-based HAART of 31 weeks [19]. The number of children in these studies with VF indicated a need of second-line antiretroviral therapy; VF mostly occurred in the first year of HAART [5,19]. Early detection of VF might allow for more second-line options. Therefore, HIV-RNA performed at 6 and 12 months after NNRTI-based HAART initiation could potentially capture the most failing children.

The sensitivity of IF in our study was low and is comparable to previous results in African HIV-infected children of 3.5% of clinical and immunologic criteria in identifying VF [13]. The criterion of IF is less sensitive to detect treatment failure compared to VF and should not be recommended [19]. However, IF criteria may continue to be used in resource-limited settings because HIV-RNA measurements are not as available as CD4 measurements.

Predictors associated with VF have been reported. Low CD4% at baseline (< 25%), and physician documentation of poor adherence were associated with VF (P < 0.05) in one study [13]. Another study reported Thai HIV-infected children receiving NVP to be 3.7 times more likely to develop VF than those receiving EFV (P = 0.006) [5]. However, we found no association of low CD4% or NNRTI type and VF in our report. This may be explained by limited number of children in our study.

Adverse events after NNRTI-based HAART commencement were reported. Using zidovudine has been related to severe anemia [25]. The proportion of children with elevated ALT grades 3-4 in our study was 1% which is lower than 3.4% in African HIV-infected children in a previous report [26]. Dyslipidemia is a common observation among HIV-infected children after HAART[27]which need the long-term follow-up.

This strength of study is the efficacy data of HIV-infected children in resource-limited setting with HIV-RNA measurement. This study has some limitation as genotypic resistance testing was not available at the time the study was conducted. There is inadequate data on the best strategy for HIV-RNA monitoring [10], therefore the future studies should be conducted to evaluate the best approaches to use HIV-RNA monitoring.

## Conclusion

In summary, NNRTI-based HAART has shown significant improvement of clinical, immunologic and virologic outcomes. At 96 weeks, 75% of children had suppressed plasma HIV-RNA and 89% had immune recovery. Almost all of VF occurred in the first year after HAART initiation. Immunologic failure had low sensitivity and should not be relied on as a monitoring for treatment failure. Plasma HIV-RNA should be performed once, and preferably twice, in the first year after NNRTI-based HAART to detect early treatment failure, and subsequently once a year if feasible as a routine monitoring for all on HAART.

## Financial disclosure and Conflict of interest

All authors declare no conflict of interest and that member of their immediate families do not have a financial interest in or arrangement with any commercial organization that may have a direct interest in the subject matter of this article.

## Authors' contributions

TB, TP, PK, and JA designed the study, collected data, wrote the first draft, reviewed and commented draft of manuscript before submission. WP, PL, CPa, and KR designed the study, collected data, reviewed and commented draft of manuscript before submission. SK, PB analyzed, reviewed and commented draft of manuscript before submission. TM and CPh collected data, reviewed and commented draft of manuscript before submission. All authors have read and approved the final manuscript.
